# Diabetes Remote Monitoring Program Implementation: A Mixed Methods Analysis of Delivery Strategies, Barriers and Facilitators

**DOI:** 10.1089/tmr.2022.0038

**Published:** 2023-03-20

**Authors:** Elizabeth B. Kirkland, Emily Johnson, Chloe Bays, Justin Marsden, Rebecca Verdin, Dee Ford, Kathryn King, Katherine R. Sterba

**Affiliations:** ^1^ Department of Medicine, Medical University of South Carolina, Charleston, South Carolina, USA.; ^2^ College of Nursing, Medical University of South Carolina, Charleston, South Carolina, USA.; ^3^ Center for Telehealth, Medical University of South Carolina, Charleston, South Carolina, USA.; ^4^ Department of Pediatrics, Medical University of South Carolina, Charleston, South Carolina, USA.; ^5^ Department of Public Health Sciences, Medical University of South Carolina, Charleston, South Carolina, USA.

**Keywords:** program evaluation, telemedicine, implementation science, qualitative research, primary care

## Abstract

**Background:**

Remote patient monitoring (RPM) is being increasingly utilized as a type of telemedicine modality to improve access to quality health care, although there are documented challenges with this type of innovation. The goals of this study were to characterize clinic delivery strategies for an RPM program and to examine barriers and facilitators to program implementation in a variety of community clinic settings.

**Methods:**

Primary data were collected via individual and small group interviews and surveys of clinical staff from South Carolina primary care clinics participating in an RPM program for patients with diabetes mellitus type 2 in 2019. We used a parallel convergent mixed methods study design with six South Carolina primary care outpatient clinics currently participating in a diabetes remote monitoring program. Clinic staff participants completed surveys to define delivery strategies and experiences with the program in a variety of clinical settings. Interviews of clinic staff examined barriers and facilitators to program implementation guided by the Consolidated Framework for Implementation Research (CFIR). Quantitative survey data were summarized via descriptive statistics. Qualitative data from interviews were analyzed in a template analysis approach with primary themes identified and organized by two independent coders and guided by the CFIR. Quantitative and qualitative findings were then synthesized in a final step.

**Results:**

RPM program delivery strategies varied across clinic, patient population, and program domains, largely affected by staffing, leadership buy-in, resources, patient needs, and inter-site communication. Barriers and facilitators to implementation were linked to similar factors that influenced delivery strategy.

**Discussion:**

RPM programs were implemented in a variety of different clinic settings with program delivery tailored to fit within each clinic's workflow and meet patients' needs. By addressing the barriers identified in this study with focused training and support strategies, delivery processes can improve implementation of RPM programs and thus benefit patient outcomes in rural and community settings.

## Introduction

Remote patient monitoring (RPM) is an increasingly utilized method of telemedicine in which data obtained at the point-of-care are transmitted for remote provider viewing and action.^1^ A growing number of health care systems currently employ RPM, with large-scale studies demonstrating effectiveness across a number of diseases.^2^ Diabetes mellitus has been the focus of many RPM interventions, with patients achieving sustained reductions in hemoglobin A1c (HgbA1c) during and after participation.^3^ The clinical benefit of RPM is maintained across diverse populations after adjusting for common social determinants of health, suggesting that RPM provides an opportunity to improve health equity.^4^ The American College of Physicians calls on providers and systems to utilize telemedicine to “enhance patient–physician collaborations, improve health outcomes, increase access to care and members of a patient's health care team, and reduce medical costs.”^5^

Despite the promise of RPM, barriers to implementation and scalability exist.^6^ Barriers exist at the levels of the patient, provider, health system, digital infrastructure, and intervention design, and these barriers vary among different populations. For example, one study found that clinics serving low-income patients report greater patient-level RPM barriers, whereas clinics serving middle-income patients report greater system barriers including challenges with program scalability and reach.^7^ Implementation challenges impede widespread use and risk continued use of small-scale inefficient RPM programs. Community-based practices are especially vulnerable to implementation barriers and yet are uniquely poised to deliver health care to vulnerable populations.

Partnerships between academic and community health centers may support RPM implementation. Such partnerships can capitalize on existing relationships between patients and their local primary care home with the resources and specialty staffing of a central RPM site. This type of arrangement has demonstrated success in statewide specialty consultation services.^8^

RPM intrinsically enables a patient–provider connection that transcends geographic and other barriers. To advance RPM program dissemination, a better understanding of barriers to implementation is needed. We report on implementation experiences from a diabetes RPM program that capitalizes on academic–community partnerships. The objectives of this mixed methods study were to (1) characterize clinic delivery strategies for an RPM program and (2) examine barriers and facilitators to program implementation in underserved and/or low-income community settings.

## Methods

### Overview

We used a parallel convergent mixed methods study design^9^ with six South Carolina primary care outpatient clinics currently participating in the Technology-Assisted Case Management in Low-income Adults with Type 2 Diabetes (TACM-2) program. These 6 parent clinics comprise 15 individual sites that were actively utilizing the program at time of this study. Practice managers were contacted by email with information about the study and to request participation. This study was approved by the Medical University of South Carolina Institutional Review Board, and a waiver of written informed consent was granted. The Consolidated Criteria for Reporting Qualitative Research (COREQ) checklist guided qualitative data methods and results reporting.^10^

### TACM-2 program and recruitment

The TACM-2 program was established in 2016 with the goal of improving chronic disease management in South Carolina. TACM-2 is a care delivery program focused on supporting diabetes and hypertension management, although in this study we focus on diabetes only because barriers to implementation are likely to vary by clinical focus. The program is based on the success of a pilot study, which demonstrated improved diabetes control among participants randomized to TACM compared with controls.^11^ TACM-2 utilizes a cellularly enabled remote monitoring device that transmits home glucose readings to a web-based secure server for provider review. Program organization, oversight, and monitoring is conducted centrally at an academic hospital, whereas local sites maintain responsibility for individual patient care and HgbA1c reporting to central data repository.

### Guiding framework

**FIG. 1.**
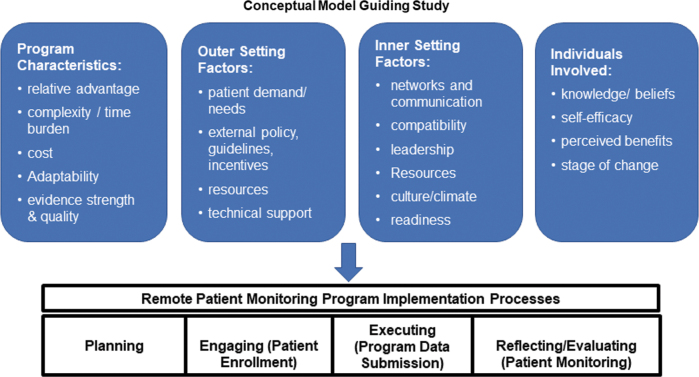
Adapted from the Consolidated Framework for Implementation Research.^12,13^

The Consolidated Framework for Implementation Research (CFIR) was used to guide data collection tools and assessment of a comprehensive set of implementation factors.^12^ This framework was developed based on existing implementation theories to guide a pragmatic approach to understanding implementation barriers, facilitators, and processes and has been widely used in chronic illness management research.^13^ The CFIR includes five main domains that can influence implementation, including individuals involved (i.e., characteristics and beliefs of those delivering and receiving the program), inner setting factors (i.e., influences within the clinic), outer setting factors (i.e., influences external to the clinic), intervention characteristics (i.e., features of the TACM-2 program itself), and implementation processes (i.e., planning, engaging, executing, evaluating).^12^ Figure 1 shows the adapted CFIR framework guiding the study.

### Data collection and measures

Basic program enrollment characteristics, including date of program initiation, number of enrolled patients, and mean HgbA1c values, were collected as part of program participation. Additional data were collected via surveys and interviews. For parent clinics with several locations, surveys were sent to all locations and responses were averaged across the sites to maintain anonymity and address missing data.

#### Site survey

A clinic representative completed an online survey assessing clinic type, staffing structure, and patient characteristics.

#### Champion survey

Program champions were identified jointly by local clinic staff and academic medical center program staff as the main advocate for the program at the local level. Some criteria considered for champion identification included being a primary contact for the program, having hands-on experience delivering the program, and length of time with program.

These champions from each clinic location completed an online survey assessing TACM-2 staffing roles and processes. This survey also assessed perceived barriers to carrying out the program (1 = not a barrier at all to 4 = major barrier) using a 12-item instrument developed for previous implementation studies.^14^ The percentage of clinics endorsing each barrier as moderate or major was calculated. Finally, we assessed perceptions concerning leadership and implementation culture using two 4-item validated scales (1 = strongly disagree to 5 = strongly agree).^15^ Due to small sample size and symmetric data, average scores (rather than median) were calculated, with higher scores indicating higher leadership and more positive culture.

#### Interviews

Individual and small group interviews were conducted with the identified clinic program champion and all available team members responsible for TACM-2 delivery and/or clinic leadership. Interviews were completed in-person or via telephone by two female investigators (E.J. and K.R.S.) with doctoral training in qualitative methods, who did not know participants. Using a semi-structured guide informed by the CFIR, participants described their clinic's patient care priorities, previous care practices, and perceptions related to the TACM-2 program including barriers and facilitators to implementation. Interviews were audio-taped and lasted 25–50 min. Strategies to assure theme saturation included using clinics that varied in program implementation processes to ensure coverage of diverse experiences, using probes to explore responses in depth, and monitoring field notes to track emerging themes.^16^

### Data analysis

Descriptive statistics were used to summarize quantitative survey data for each clinic. Transcriptions of interviews were analyzed using NVivo software (QSR International, 2020).^17^ A template analysis approach was used with an initial codebook guided by the CFIR also allowing new codes to be generated directly from the data.^17–19^ An iterative process was used with two independent coders. The codebook evolved over time by refining code definitions, collapsing a set of codes, and adding several new codes, while maintaining an audit trail of the process. Coders read and reread each transcript, organizing primary themes and resolving discrepancies in group meetings.^20,21^ Results from each source were summarized and compared to identify similarities and differences. The final analysis step involved synthesis of quantitative and qualitative findings.

## Results

### Clinic characteristics

Six clinics, with 15 unique practice sites participating in TACM-2, were invited to participate in qualitative and quantitative study elements. Five of six parent clinics completed champion and site surveys. We conducted 10 individual and small group interviews (*N* = 20 participants overall; range number of participants 1–8) with each clinic represented (*n* = 6).

Clinic characteristics and patients varied widely (Table 1). Participants highlighted consistently positive perceptions of leadership (mean score 4.15; standard deviation [SD] 0.49; range 3.5–5) and a positive implementation climate (mean score 4.4; SD 0.55; range 4–5) on champion surveys.

**Table 1.** Clinic Structural, Practice, and TACM-2 Program Characteristics

	Clinic 1	Clinic 2	Clinic 3	Clinic 4	Clinic 5	Clinic 6
Clinic characteristics						
Type	Free	Free	Free	FQHC	FQHC	Academic medical center
No. of sites	1	1	2	5	6	1
Clinic staffing						
Full-time equivalent MD or DO	<1	0	0	*Missing*	16	12
Full-time equivalent PA or NP	<1	1	2	*Missing*	10	0
Full-time equivalent RN	<1	1	0	*Missing*	23	2.5
Pharmacist on-site	Yes	No	Yes	No	Yes^a^	Yes
Diabetes educator on-site	Yes	Yes	Yes	No	Yes^a^	Yes
Average number of patients scheduled per day, per one provider (MD, DO, PA, or NP)	*Missing*	≤16	≤16	*Missing*	17 to 22	≤16
Annual staff turnover rates	<10%	<10%	10–25%	—*(Missing)*	26–50%^b^	10–25%
Patient demographics						
Total number of patients served by clinic	500–1000	2000–3000	<500	8000–9000	>30,000	5000–6000
Non-English speaking	70%	60%	<5%	<5%	<5%	<5%
Race of patient population						
White or Caucasian	10%	11%	15–23%	18%	61%	30%
Black or African American	10%	12%	77–85%	74%	21%	67%
Hispanic or Latinx	78%	76%	<1%	5%	15%	1%
Asian, Native Hawaiian, Pacific Islander, American Indian, Alaskan Native	2%	1%	0%	2%	1%	1%
Other	<1%	<1%	<1%	1%	2%	2%
Most common insurance	None/uninsured or self-pay	None/uninsured or self-pay	None/uninsured or self-pay	Medicaid	Medicaid	Medicare, commercial

Support staff: registration or clinic support staff.

^a^ Only three of six sites have on-site pharmacists and diabetes educators.

^b^ Three of six sites said 26–50%, two sites said 10–25%, and one site said <10%.

DO, doctor of osteopathic medicine; FQHC, Federally qualified health center; MD, doctor of medicine; NP, nurse practitioner; PA, physician assistant; RN, registered nurse; TACM-2, Technology-Assisted Case Management in Low-income Adults with Type 2 Diabetes.

### Program organization and delivery strategies

Table 2 provides quantitative and qualitative results associated with key delivery elements: patient enrollment, data submission, and monitoring. Interview participants described using an iterative process to identify the best delivery strategy for TACM-2, largely influenced by clinic staffing, infrastructure, and resources. Interviews and surveys highlighted heterogeneity in program organization and staffing models: some had one dedicated person in charge of all program procedures, whereas others used a team approach with all staff participating.

**Table 2.** Clinic TACM-2 Program Delivery Processes: Quantitative Data and Supporting Quotations

	Clinic 1 (***n*** = 3)	Clinic 2 (***n*** = 1)	Clinic 3 (***n*** = 3)	Clinic 4 (***n*** = 2)	Clinic 5 (***n*** = 8)	Clinic 6 (***n*** = 3)
Enrollment processes						
Date of first enrolled patient	Spring 2017	Summer 2017	Spring 2018	Summer 2018	Spring 2018	Fall 2017
Enrolled patients, *n*^a^	53	15	69	62	508	220
Mean baseline A1c	10.6% (92 mmol/mol)	10.7% (93 mmol/mol)	10.6% (92 mmol/mol)	10.6% (92 mmol/mol)	11.1% (98 mmol/mol)	10.1% (87 mmol/mol)
Type of staff member performing the following roles						
Screening or identifying eligible patients	RN	Physician	CMA or LPN	*Missing*	APP	Physician
Enrolling patients	RN	Support staff	CMA or LPN	*Missing*	Support staff	RN
Training patients on device	RN	Support staff	CMA or LPN	*Missing*	Support staff	RN
Location for enrollment	Examination room	Examination room	Nurses station	*Missing*	Conference room, examination room, nursing supervisor's or CDE's office	Conference room
	“Once the patient is diagnosed with diabetes, which has been pretty often, … we explain to them how—what diabetes is. And then we provide, if they qualify, we give them the meter, we show them how to use it. But we also give them general information about healthy eating, the importance of general checkups, the importance of taking medications, lifestyle changes. So, we give them information in their language.” (clinic 2)					
	“What I've seen here at the office is whenever the patient comes in for their three month or six month diabetic visit, the provider will check to see if they're already checking their blood sugar, if they have insurance that covers that or even if they do or they don't, just the simple fact that we're able to monitor their blood sugar through your system gets some of the providers into the habit of having them go ahead and setting up with a TACM machine before they leave our office.” (clinic 5)					
	“When we're signing people up we should have a contract that says this is what is expected of you: A1cs [drawn] at 6 months and 12 months, you to be checking your sugar. It's a contract. We're giving you this, these are all the fun things. This is what is expected of you to do it. And to have people to agree to that up front.” (clinic 5)					
	“We have a person in our clinic that does all the data sorting, he will create the list of patients that have had an A1C in our clinic over eight, and that list will be sent to the resident. They will identify who needs to be called because they will view the patient's chart for any exclusions.” (clinic 6)					
	“I came up with a system, where we identified all of our diabetic patients through the EMR and then flagged them and reached out to them and then once I saw them, made sure that they had consistent follow up and those sorts of things.” (clinic 1)					
Data submission processes						
On-site laboratory presence	No	No	No	Yes	Yes	Yes
Location for follow-up visits	Examination room	Examination room	Nurses station	*Missing*	Conference room, examination room, nursing supervisor's or CDE's office	Examination room
6-Month data submission rates	65%	50%	75%	65%	72%	97%
12-Month data submission rates	55%	25%	40%	23%	60%	94%
Enrolled patient outcomes						
Mean A1c reduction at 6 months	1.7% (18 mmol/mol)	1.5% (16 mmol/mol)	1.8% (19 mmol/mol)	1.3% (14 mmol/mol)	2.2% (24 mmol/mol)	1.5% (16 mmol/mol)
Mean A1c reduction at 6 months	1.1% (12 mmol/mol)	0.4% (4 mmol/mol)	1.5% (16 mmol/mol)	0.8% (8 mmol/mol)	2.1% (23 mmol/mol)	1.3% (15 mmol/mol)
	“Our nurses … were used to doing the paper. So when they got the internet, they submitted it and then something would be missing in that patient, blood pressure, that patient account number. So I think the paper worked better than the internet.” (clinic 4)					
	“I don't have any concerns as far as the portal. It's easy to go in there and update any kind of information. It's user friendly. The way we're submitting the data now is so much better than having to fax it. Like I said, the e-mails, the monthly [reminder] e-mails on what patients are needing is very, very helpful.” (clinic 5)					
	“As far as following up with patients, when I first started, I think that the TACM program would send over the monthly reports … of who had essentially been lost to follow up, you know, who we didn't have a 6 month or 12 month A1C on, and there was a huge, huge gap, there. And that's [our] clinic's issue. So, we cleaned that up significantly and made sure we had patients following up regularly and getting their labs. So, that was clinic side stuff.” (clinic 1)					
	“The transportation, the lack of ability to reach [patients]. If they don't have a lot of family or friend support, they tend to be less compliant than most [with follow-up]. And some of them, not a whole lot, don't quite understand. Maybe they're young or they just don't grasp the concept of how well this is going to help them, so there's that barrier as well.” (clinic 5)					
Patient monitoring processes						
Type of staff member performing the following roles						
Monitoring blood glucose data	RN	Support staff	APP	*Missing*	APP	Physician
Calling patients to make adjustments	CMA or LPN	Support staff	APP	*Missing*	Support staff	Physician
	“And so my role after receiving the list of patients to go through is to review their postings of their glucose log and their blood pressure log and then review what medications they're on, come up with my own clinical plan, so what I'd like to do in terms of medication adjustments.” (clinic 6)					
	“… once they're in the system, the provider will review their uploads and all their glucose testing that they do throughout the week and then they, once they're coming for their follow up, then they'll look at their readings and see if they've had any spikes or are they getting stable or consistent and educate them accordingly on medication or diet, exercise, and so on.” (clinic 1)					
	“At first, it's like, ‘Oh man.’ It is a lot of responsibility and you've got to do it and find a way to manage the patients every two weeks because we would get the reports from MUSC, but the supervisors were finding that … pulling the reports ourselves, we could get it faster and quicker.” (clinic 5)					
	“But I do know that we had some major issues. Like, I mean, month delay getting test strips and lancing devices and stuff like that.” (clinic 1)					
	“I mean whatever the patients weren't uploading, we would call them. We would have them bring their machines. If there was any troubleshooting that needed to be done.” (clinic 5)					
	“Often because these patients are complex, we end up pulling in the in-clinic pharmacist as well to assist with some of this medication change decisions.” (clinic 6)					
	“So having the machine where they didn't have to pay for it, but it also uploaded to the cloud where we could monitor them really was a saving grace. It allowed us to keep up with our patients in between visits, see how their blood sugars were running, do any medication changes. It was very helpful for the patients.” (clinic 5)					
	“I think we've seen such a dramatic improvement in the patient care and the quality of the care that we can provide … So, we absolutely are appreciative of this program.” (clinic 1)					

APP, advanced practice provider; CDE, certified diabetic educator; CMA, certified medical assistant; LPN, licensed practical nurse; RN, registered nurse.

^a^ As of March 31, 2020.

#### Enrollment

Procedures included identification of patients with a qualifying HgbA1c via embedded electronic medical record screening or in-office laboratory and/or chart review. Following identification, consenting patients were typically enrolled and provided education on the TACM-2 device the same day. Enrollment paperwork and device training took ∼30 min with variability based on patient need (e.g., clinical, translation, or education needs; Table 2).

A variety of clinic staff members participated in enrollment processes, with nursing and support staff most commonly enrolling and training patients. Staff turnover varied among clinics and impacted training needs for enrollment.

#### Data submission

TACM-2 data submission processes included the submission of enrolled patient HgbA1c data at baseline, 6 and 12 months through a web-based portal. Some staff voiced the convenience of this system, whereas others preferred a fax system. Several mentioned the benefits of receiving reminders from the program. Data submission was challenged by lack of on-site laboratory services and patient failure to follow-up.

#### Patient monitoring

Clinic TACM-2 patient monitoring processes included managing supply needs, review of program-generated reports flagging patients with high blood sugars, direct blood sugar review in the portal, calling patients not regularly monitoring, and using data to guide medication changes. As with other TACM-2 processes, clinics appeared to have varied staffing models and resources (e.g., certified diabetes educators and/or pharmacists) to support patient monitoring. Some clinics only retrieved data to use as ancillary data during office appointments, whereas other clinics used data in real time or between visits to support additional follow-up. While some clinics were highly satisfied with program communication and receipt of supplies and data to monitor patients, others complained about delays. The majority of clinics reported that the program improved quality of care by providing increased communication and monitoring between the clinics and patients.

### Implementation barriers and facilitators

Surveys and interviews identified barriers and facilitators to the implementation of the TACM-2 program. Surveys highlighted that few barriers were perceived to carrying out the program. Specifically, financial resources, staff commitment, evidence about the program's value, leadership, space, communication within the clinic and with central program staff, and staff training were unanimously considered only a minor barrier or not a barrier at all (*N* = 5). The following factors were considered a major or moderate barrier in only one clinic each: having designated staff to coordinate, other issues being higher priority and information technology. The top barrier was time, endorsed by three of five (60%) of clinics. Interviews identified unique themes representing barriers and facilitators to implementation (Table 3).

**Table 3.** Focus Group Themes: Barriers and Facilitators to Implementation of a Remote Patient Monitoring Program

Theme	Definition	Exemplary Quotations
Intervention characteristics		
Relative advantage	Stakeholders' perceptions of the advantage of implementing the program vs. an alternate solution or usual care.	“I can say very generally that meter supplies are expensive. So, that's certainly a barrier for patients as far as glucose monitoring and frequency … certainly, the program helped with that, keeping on top of that.” (clinic 1) “It creates an atmosphere where we don't have to worry about patients bringing in a log of blood sugars, or trying to troubleshoot a glucometer. We can just go online and download the data that's available.” (clinic 3)
Complexity	Perceived difficulty/ease of implementation, reflected by duration, scope, disruptiveness, and number of steps.	“It was very simple. We loved the use of technology. Like I said, it made our lives much easier.” (clinic 1) “You know troubleshooting those machines is difficult. So having somebody else reach out to them when there was technical problems, that was very helpful.” (clinic 5)
Outer setting		
Patient demand/needs	The extent to which patient demand and service needs exist; clinic awareness and prioritization of patient needs and barriers to meeting those needs.	“We, as you know, as an FQHC, we are under water all the time, trying to provide services for our patients and resources are limited, so when we do have opportunities, we tend to only go for the opportunities that will lead to the biggest impacts for our patients, which is why we're doing this program. So that is one of our priorities … our staff, they wear so many hats as it is and so this is just another thing, but the need is greater than the pain of it.” (clinic 5) “So, we have a fairly complex group of patients that are medically complex that in turn are also fairly socially complex. I think we have a pretty high percentage of Medicaid population and a population that is a fairly high use of medical resources due to their medical and social complexity.” (clinic 6) “But patients come here because they cannot afford sometimes to even pay for medications, so they couldn't afford to buy a glucometer. So, a lot of them will stay uncontrolled and they will just come here and find out how bad their blood sugar was. Even, you know, only when they would come here.” (clinic 2) “Overall, when the patient comes in, especially because they come from low poverty as well as their educational background, they receive it very happily. They are very grateful in getting the supplies that they need free of cost, because it's such a burden on them. And if they have to choose whether I buy a monitor or I supply something else for the family, then they're not going to purchase the monitor or their medications or go see a doctor, for that matter.” (clinic 1)
External policy/guidelines and incentives	External strategies to spread and sustain programs, including policy and regulations, external mandates, and guidelines.	“The quality improvement initiatives often come from any sort of grant funds. It is a health clinic, mainly run on donations and any sort of grants. They're going on all the time. So, the South Carolina Free Health Clinic Association … they would essentially put forth quality measures.” (clinic 1) “Because the fact that we are with the South Carolina Free Clinic Association. And we do have to monitor blood pressures and A1Cs, like consistently reporting about it. So, this device has been so helpful for us to pull those numbers all together. So now that we can work with your portal, it is so much more helpful.” (clinic 3)
Program–clinic partnerships	Telemedicine program operational style, partnership-building strategies, and interactions around training and support provision for program implementation.	“I think they really did all the hard work, the footwork, they had the paperwork, any questions we had, they got back to us. We did at one time have a lot of hard time with the equipment. They were able to troubleshoot and send us information to help us troubleshoot to get the meters running and connecting the way they should have. They were just very supportive. I don't know that I would have asked that they did more.” (clinic 5) “No challenges. The only thing I did see is that it seemed like we—once we got the program, everything fell on us. We had to monitor the patients. We need to contact the patient. And that became a barrier because I thought for me learning that they would come out and help more, but they didn't.” (clinic 4) “But it would be nice if locally if there's somebody else around here using the [program] like us, maybe to help staff stay in compliance themselves, maybe if they had another person in this area they can talk to, and go sit down with, or they can come over here, and they can network more and see how successful their clinic is, versus ours. And that would be wonderful to have that kind of support, as well.” (clinic 3) “So, again, that stuff might have been communicated, but it just feels like the support drops off for patients who are out of that 12 month window. Which is fair … but yeah, we were trying to make arrangements to continue access for our patients who have gotten used to something. I think overall if we could have in person meetings …, quarterly meetings, that would be great.” (clinic 1)
Inner setting		
Networks and communication	The nature and quality of webs of social networks and formal and informal communications between leaders, nurses, physicians, and staff within an organization.	“It's every nurse, every nurse, all hands on deck, running the reports, giving them to the doctors, calling the patients. So it's a lengthy process, but we had staff wanting to help the patients, for sure. Nobody was like, ‘Oh no.’ It is tedious and it's time consuming for the staff, but we do what we have to do to make it work.” (clinic 5) “You just have to take on a little bit more. But once you get it in and you get it structured, it's pretty much easy. But just to get a staff to buy in sometimes has been very difficult.” (clinic 3) “[The Coordinator and Nurse] work closely together to make sure I'm available when the patient's going to come …. we also are very closely connected—he has to know how many devices I have in stock before he can call people. So we have to kind of go back and forth.” (clinic 6)
Compatibility	The degree of tangible fit between the intervention and staff norms, values, and perceived risks and needs, and how the intervention fits with existing workflows and systems.	“So initially, three years ago, when [program staff] came in to explain the process to us, initially, we were really not confident that our patients would be able to do it because of the low literacy level, and explaining to them the Internet—because most of our patients, they don't have a phone line or they don't have the computer, they're not very computer savvy.” (clinic 1) “Well, the program itself, TACM is not the only thing we are doing. And when we have to work in somebody else's system it's like getting out of our system and working in someone else's system. Which tends to be, a lot more work.” (clinic 5) “It—well, when I first started TACM, I felt like it would have fit into our busy clinic, because it helped with the patients who were greater than … A1Cs greater than eight. But from being in the program, I realized that it's kind of put like a barrier on our program because those patients are not able to get the measures over their internet because they don't have Wi-Fi.” (clinic 4) “I think it fits well. The vision of our clinic; the vision and mission is to improve the life of individuals in [our county]. And being that we serve patients who are uninsured, a program that provides the means for them to take control and monitor their diabetes status at no cost to them aligns perfectly with the vision, and values, and the work flow of the clinic.” (clinic 3)
Leadership	Commitment, involvement, and accountability of leaders and managers with implementation; presence of site champion.	“As far as clinic level leaders, I was the only primary care provider, so that was kind of it. And then [the administrator] is the head of the clinic, so she was pretty removed for the process, but certainly supportive.” (clinic 1) “We've had the CEO supporting the program from the very beginning, including our CMO, they've always been supportive … Our CEO has been involved with it. Our pharmacy's been involved with it. It's just kind of embedded in us now all the way around at every site in the state.” (clinic 5) “Our [Center Director] is our interim director. So he's our direct boss and then the other people working with us on this are [two clinician leaders]. And those three people equally, they are on all of our calls, our weekly staff meetings. They've all gone with us on site visits. They've been fantastic.” (clinic 6)
Resources	The level of resources dedicated for implementation and ongoing operations, including money, training, education, physical space, staffing, and time.	“Yes. The biggest, biggest, biggest challenge is the diabetes educator, you know? So, we tried several different options. First, we had a volunteer diabetes educator, and she was great—but again, because of her limited communication ability [in Spanish], she got a little frustrated because, again, the patients would not understand. And she did classes and she tried to do all those things, but I think it was just a little overwhelming for her, too.” (clinic 1) “In brief, I know from the clinician side, we are very fortunate to have a direct resource in an in-clinic pharmacist on a daily basis who is also heavily involved in the TACM programs and will alternate with the providers seeing these patients or calling these patients. And so they're an in-house resource that kind of is within our work flows for making changes or running things by—from a clinician side, that's extremely helpful.” (clinic 6) “I feel like we have the staff and our board who were willing to make it work and make it happen regardless of what we had to do. As far as space goes, I feel like each supervisor of the nursing staff, they all had space in their offices to keep the supplies put up and the machines and those sorts of things. Our biggest challenge was our manpower, but it didn't mean that we didn't want to help.” (clinic 5) “And just getting one [staff] person to get excited about it and do it, and then something happens. That person has to retrain, retrain. And I want to say we retrained three—this will probably be the fourth—this will be the third person on the device that has to be trained. That's been our biggest challenge. Just trying to get somebody to stay, just to get that program running.” (clinic 3)

#### Relative advantage (intervention characteristics)

The degree to which participants perceived that the TACM-2 program was an advantage over previous care practices and how these beliefs impacted implementation was mixed. Two free clinics specifically described that the program offered an advantage, as it provided glucose monitoring resources and supplies at no cost, while also offering convenience of tracking patient data from home. However, one clinic perceived that the program was not advantageous to patients based on a misunderstanding of the program's required communication platform (internet vs. cellular access).

#### Complexity (intervention characteristics)

Limited feedback was provided regarding the complexity of the TACM-2 program and how this impacted implementation. One free clinic and one Federally Qualified Health Center (FQHC) described the TACM-2 program as simple, user-friendly, and easy to implement; yet, staff from a different clinic reported difficulties in troubleshooting the devices. Another FQHC site commented on the value of patient-directed technical support.

#### Patient demand/needs (outer setting)

Patient demand and clinic commitment to address uncontrolled diabetes represented key drivers of implementation. All clinics reported high demand for a program such as TACM-2. Due to high rates of diabetes, lack of insurance, and poverty in the rural and underserved communities, patients experience barriers to blood glucose monitoring that TACM-2 addresses, including expense of testing supplies and medications. One FQHC clinic considered the pros and cons of investing time and resources into programs, ultimately committing to TACM-2 due to high patient demand and promising benefits.

In parallel to high patient demand, five clinics perceived that patients were grateful for TACM-2 as it supports diabetes management and provides supplies not otherwise accessible. Participants from these five clinics observed that through the program, patients learned to take ownership of their diabetes and how to identify abnormal glucose levels. Over time, patients valued being able to see their HgbA1c levels decrease.

Although TACM-2 was perceived to meet patient demand and improve patient satisfaction, participants within all clinics still noted implementation barriers as some patients lacked transportation for enrollment and follow-up visits. Low educational attainment, low literacy levels, and language barriers were described as challenges to device training. In addition, some clinics perceived that access to technology (cell phones, internet access, comfort with technology) represented communication barriers that impacted program feasibility, representing a true digital divide.

#### External policy/guidelines (outer setting)

Several external factors, including diabetes management and reporting guidelines, were considered influential to TACM-2 delivery. Various guidelines were followed for quality initiatives and care goals, and two free clinics directly described how the program facilitated adherence to quality initiatives (e.g., HgbA1c levels). The TACM-2 portal for data monitoring and reporting allowed clinics to efficiently aggregate data for reporting.

#### Clinic–program partnerships (outer setting)

There were mixed perspectives about partnerships between clinics and the academic medical center and their influence on program implementation. Five clinics reported timely and effective communication with the program during early implementation and start-up processes. These clinics appreciated the detailed easy-to-follow setup steps as well as ongoing support for troubleshooting, technical assistance, and reporting.

Challenges establishing partnerships were defined by one of the clinics, typically during program setup and abated by identification of a clinic champion and clear delineation of clinic responsibilities. Suggestions to improve program–clinic partnerships included quarterly in-person training and monitoring, connections to other clinics implementing TACM-2 for networking, and more support and resources as patients' transition off the 12-month program.

#### Networks and communications (inner setting)

Teamwork and communication within the clinic were viewed as important to program delivery, but clinics varied in the ways they assembled teams around the program. Respondents commonly reported that it took time to establish implementation practices after trialing varied roles and strategies. Regardless of the approach taken by teams, all clinics highlighted the importance of communication for timely completion of program steps. Some successful clinic strategies described for improving communication and teamwork included consideration of varied models to identify best practices and holding routine team meetings to discuss program issues.

#### Compatibility (inner setting)

Perceptions about program compatibility were important to implementation. Reflections included staff perceptions about the suitability of the program to address patient needs as well as consideration of the program's fit within clinic workflows. Generally, respondents emphasized that the program was a good match as it focused on meeting high-risk patients' needs, although a concern was raised about technological access and literacy.

Two clinics appreciated that the program streamlined current workflows (facilitating review of data at appointments) and offered additional support (program oversight of patient data). In contrast, a different site highlighted inconsistency between program and clinic practices (patient identification, treatment algorithms), which added work and was disruptive.

#### Leadership (inner setting)

While clear leadership for the program was not explicitly described as a driver of successful program implementation, all sites did identify a program leader and described their varied roles. Leadership structure and involvement varied among sites with most sites having one primary leader and a few sites having a more collaborative leadership team. The majority of leaders were on-site, with some holding administrative roles and others with clinical roles.

#### Resources (inner setting)

Resources such as staffing, space, and supplies were considered important to the program and facilitated successful delivery. As described above, clinics had varied models of staffing to support and reinforce the program. Clinics frequently described the burden of program-related tasks in addition to other job responsibilities. Having a small number of staff and high rates of turnover were reported as common challenges to TACM-2 delivery in all free clinics and FQHCs, and retraining efforts drained clinic resources. A few clinics mentioned the need for materials and staff to communicate with Spanish-speaking patients. Suggestions to improve patient engagement from individual clinics included direct technical support, educational materials and support in Spanish, and patients' ability to log on and monitor blood sugar numbers themselves.

## Data Synthesis

Quantitative data showed that clinic, patient, and program delivery characteristics varied in TACM-2 clinics. Qualitative data offered additional insight into implementation barriers and facilitators. Survey data showed variability in resources available to support program delivery, and interview data highlighted that appropriate staffing models as well as infrastructure were supportive of the program. Both survey and interview data highlighted time as a key barrier to program implementation. Interviews also highlighted high staff turnover rates as a challenge, causing staff shortages, a need for increased training, and further increasing time demands. Qualitative data also described patient resource barriers including transportation as well as language and knowledge barriers hindering program understanding and the ability to carry out program demands at home.

Surveys demonstrated positive perceptions of leadership in clinics, and interviews revealed that while all sites did identify a leader, there were different leadership styles among clinics that may have influenced implementation. Participants appeared to perceive the presence of a collaborative leadership team as facilitating implementation processes. Surveys revealed positive perceptions of the implementation climate for delivery of the program, but interviews highlighted variability in this area with only some clinics reporting positive communication and teamwork practices. Interviews additionally revealed that positive collaborations between the clinics and the academic center contributed to productive implementation climates. High patient demand, or high rates of diabetes in clinic communities, and clinic commitment to help patients were the main facilitators to implementation.

## Discussion

RPM has been increasingly utilized in clinical practice for patients with diabetes and has demonstrated improved patient outcomes.^3,4,22^ However, there have been challenges to widespread dissemination of this multifaceted complex innovation.^6,22^ This CFIR-guided mixed methods study addressed this research–practice gap and utilized implementation science methodologies^12^ to examine program delivery strategies and common implementation barriers and facilitators to delivery of a diabetes RPM program in underserved community settings.

Delivery strategies varied widely across clinics as they had diverse workflows, staffing and leadership models, and practical support to maintain the program. This variability highlights the need for RPM programs to be flexible and the feasibility of delivering programs in varied settings. Results also identified barriers and facilitators at the clinic, patient, and program levels.

At the clinic level, *leadership buy-in* emerged as important to program implementation. However, despite strong endorsement of leadership for the program, practical barriers had to be overcome to facilitate progress. Specifically, *staffing* was influential to program delivery, as has been documented in RPM studies of other chronic diseases.^23^ Free clinics generally relied on more volunteer staff, whereas academic clinics relied more heavily on trainees and resident physicians.

All sites struggled with the impact of staff turnover, which demanded additional time for training and programmatic inefficiencies. This could lead to lower program enrollment, less consistent monitoring, less fidelity in process measurement, and diversion of resources to accommodate repeated trainings. Importantly, staffing time increased to meet patient needs, including lower health literacy, lack of diabetes awareness, and non-English-speaking language preference. This may have stretched staffing, increased program-specific burnout, and/or limited available resources to engage other patients.

Several clinics described *multidisciplinary* resources embedded within the clinics, including on-site laboratories, pharmacists, and clinical diabetic educators (CDEs) who provided ancillary support and education. By sharing program responsibilities with other staff members, multidisciplinary clinics may have faced less strain on staffing and time. Additionally, the added educational support and convenience of completing blood draws on-site may influence a patient's likelihood to remain engaged in the program. Further investigation into the impact of multidisciplinary resources on program engagement is important.

Patient factors influenced program delivery as clinics tailored implementation to meet their needs. The free devices and monitoring were seen as a benefit for low-income and uninsured patients. As such, clinics serving low-income populations may be more likely to invest in the program, and patients may be more likely to engage. Contrarily, a large non-English-speaking population may be a barrier to implementation owing to greater staffing demands, as described above. Patients lacking stable housing, transportation, and insurance face barriers to program retention and follow-up data collection, a metric by which clinics were measured. A tailored implementation approach accounting for needs of the target population is important, as demonstrated by our work and that of others.^24^

Program factors, including local-central communication patterns and comfort with the data reporting system, impacted program delivery and were represented in reported barriers and facilitators to implementation. Program-level barriers to RPM implementation are well documented and include cost, poor integration with current workflows, data overload, and increased workload for providers.^23–26^ In our study, most clinics communicated frequently with the central site and received timely supply shipments, thereby facilitating program implementation.

For clinics well versed in online monitoring and data reporting, the program's online reporting system was viewed as beneficial, increasing clinics' abilities to achieve reliable convenient data exchange between patient and provider. Alternatively, some clinics found the program to be taxing on staff and disruptive to usual workflow. Collaboration between academic medical centers and community partners has been accomplished in other studies, as in ours, and has been emphasized as an important tool for disseminated care.^8,27^ Our qualitative data demonstrate the importance of clear expectations and roles, with ambiguities threatening program success.

Results highlight the promise of RPM programs in community settings and potential support strategies needed at the clinic, patient, and program levels for implementing RPM programs. Similar to findings in a recent qualitative study of mobile health technologies, we found complex relationships of clinical staff with telehealth programs, specifically regarding inter-site collaboration, care delivery efficiency and flexibility, financial and technological barriers, training requirements, and patient needs and skills.^28^

A rigorous planning phase could support the assessment of clinic readiness and patient needs to guide tailored practices for staffing and workflows to set clinics up for success. Building clinic teams, assigning roles and responsibilities, establishing communication preferences, and providing training will also help teams prepare for implementation. Clear messaging in the planning phase can help define expectations to prepare to meet patient needs. More work is needed to guide development of protocols for ongoing training, technical assistance, and troubleshooting challenges to support programs over time.

Despite the strengths including use of the CFIR to guide examination of a comprehensive set of implementation factors and our mixed methods approach, there are several limitations of this work. First, findings are subject to nonresponse bias as not all participants responded to all portions of the study. A strength, however, is that all sites were represented in interviews to allow comprehensive qualitative perspectives. Second, the perspective of patients is not captured and should be a focus of future studies, although work in the cardiovascular field suggests that patients appreciate the benefits of RPM.^23^ Third, the program under study is implemented within a single state, and thus, generalizability is limited. There are differences in state regulations related to RPM across states, including insurance payor variations, which were not assessed in this study.

## Conclusions

Clinic, target population, and program elements impact RPM program delivery, but such programs can be adapted for community clinics with varied structures. Clinical staff perceive a number of barriers to program implementation that can be mitigated with training and support strategies, such as a structured planning to accommodate unique clinic staffing and clinic workflows, support for team-building within clinics, and development of tailored protocols for training, program delivery, and support. Despite implementation challenges, RPM programs can capitalize on clinical staff's motivation and commitment to help patients^28^ to improve patient outcomes. Future research is needed to expand the current study's findings to other geographic areas, which will allow descriptions of differences across state populations and with regard to differences in state regulations and payor variations. Future studies should also evaluate planning, training, team building, and implementation strategies to support program sustainability.

## Abbreviations Used

CDEs: clinical diabetic educators
CFIR: Consolidated Framework for Implementation Research
COREQ: Consolidated Criteria for Reporting Qualitative Research
HgbA1c: hemoglobin A1c
RPM: remote patient monitoring
TACM-2: Technology-Assisted Case Management in Low-income Adults with Type 2 Diabetes

## References

[1] Center for Connected Health Policy. Remote patient monitoring. Available from: https://www.cchpca.org/topic/remote-patient-monitoring [Last accessed: October 24, 2021].

[2] Farias FAC, Dagostini CM, Bicca YA, et al. Remote patient monitoring: A systematic review. Telemed J E Health 2020;26(5):576–583; doi: 10.1089/tmj.2019.0066 31314689

[3] Zhai YK, Zhu WJ, Cai YL, et al. Clinical- and cost-effectiveness of telemedicine in type 2 diabetes mellitus: A systematic review and meta-analysis. Medicine (Baltimore) 2014;93(28):e312; doi: 10.1097/MD.0000000000000312 25526482 PMC4603080

[4] Kirkland EB, Marsden J, Zhang J, et al. Remote patient monitoring sustains reductions of hemoglobin A1c in underserved patients to 12 months. Prim Care Diabetes 2021;15(3):459–463; doi: 10.1016/j.pcd.2021.01.005 33509728 PMC8131229

[5] Daniel H, Sulmasy LS. Health and Public Policy Committee of the American College of Physicians. Policy recommendations to guide the use of telemedicine in primary care settings: An American College of Physicians position paper. Ann Intern Med 2015;163(10):787–789; doi: 10.7326/M15-0498 26344925

[6] Klonoff DC. The current status of mHealth for diabetes: Will it be the next big thing? J Diabetes Sci Technol 2013;7(3):749–758; doi: 10.1177/193229681300700321 23759409 PMC3869144

[7] Alvarado MM, Kum HC, Gonzalez Coronado K, et al. Barriers to remote health interventions for Type 2 Diabetes: A systematic review and proposed classification scheme. J Med Internet Res 2017;19(2):e28; doi: 10.2196/jmir.6382 28193598 PMC5329647

[8] Lesher AP, Fakhry SM, DuBose-Morris R, et al. Development and evolution of a statewide outpatient consultation service: Leveraging telemedicine to improve access to specialty care. Popul Health Manag 2020;23(1):20–28; doi: 10.1089/pop.2018.0212 31161963

[9] Palinkas LA, Aarons GA, Horwitz S, et al. Mixed method designs in implementation research. Adm Policy Ment Health 2011;38:44–45; doi: 10.1007/s10488-010-0314-z 20967495 PMC3025112

[10] Tong A, Sainsbury P, Craig J. Consolidated criteria for reporting qualitative research (COREQ): A 32-item checklist for interviews and focus groups. Int J Qual Health Care 2007;19(6):349–357; doi: 10.1093/intqhc/mzm042 17872937

[11] Egede LE, Williams JS, Voronca DC, et al. Randomized controlled trial of technology-assisted case management in low income adults with Type 2 Diabetes. Diabetes Technol Ther 2017;19(8):476–482; doi: 10.1089/dia.2017.0006 28581821 PMC13170820

[12] Damschroder LJ, Aron DC, Keith RE, et al. Fostering implementation of health services research findings into practice: A consolidated framework for advancing implementation science. Implement Sci 2009;4(1):50; doi: 10.1186/1748-5908-4-50 19664226 PMC2736161

[13] Kirk MA, Kelley C, Yankey N, et al. A systematic review of the use of the Consolidated Framework for Implementation Research. Implement Sci 2016;11:72; doi: 10.1186/s13012-016-0437-z 27189233 PMC4869309

[14] Sterba KR, Johnson EJ, Nadig N, et al. Determinants of evidence-based practice change uptake in rural intensive care units: A mixed methods study. Ann Am Thorac Soc 2020;7(9):1104–1116; doi: 10.1513/AnnalsATS.202002-170OC PMC7722472 32421348

[15] Fernandez ME, Walker TJ, Weiner BJ, et al. Developing measures to assess constructs from the Inner Ssetting domain of the Consolidated Framework for Implementation Research. Implement Sci 2018;13(1):52; doi: 10.1186/s13012-018-0736-7 29587804 PMC5870186

[16] Saunders B, Sim J, Kingstone T, et al. Saturation in qualitative research: Exploring its conceptualization and operationalization. Qual Quant 2018;52:1893–1907; doi: 10.1007/s11135-017-0574-8 29937585 PMC5993836

[17] NVivo Software. NVivo Qualitative Software. QSR International [serial online]; 2020. Available from: https://www.qsrinternational.com/ [Last accessed: December 15, 2020].

[18] Crabtree B, Miller W. Using Codes and Code Manuals: A Template Organizing Style of Interpretation. Sage: Newbury Park, CA; 1999.

[19] Brooks J, McCluskey S, Turley E, et al. The utility of template analysis in qualitative psychology research. Qual Res Psychol 2015;12:202–222; doi: 10.1080/14780887.2014.955224 27499705 PMC4960514

[20] Korstjens I, Moser A. Series: Practical guidance to qualitative research. Part 4: Trustworthiness and publishing. Eur J Gen Pract 2018;24(1):120–124; doi: 10.1080/13814788.2017.1375092 29202616 PMC8816392

[21] Nowell LS, Norris JM, White DE, et al. Thematic analysis: Striving to meet the trustworthiness criteria. Int J Qual Methods 2017;16:1–13; doi: 10.1177/1609406917733847

[22] El-Rashidy N, El-Sappagh S, Islam SMR, et al. Mobile health in remote patient monitoring for chronic diseases: Principles, trends, and challenges. Diagnostics 2021;11:607; doi: 10.3390/diagnostics11040607 33805471 PMC8067150

[23] Maines M, Tomasi G, Moggio P, et al. Implementation of remote follow-up of cardiac implantable electronic devices in clinical practice: Organizational implications and resource consumption. J Cardiovasc Med (Hagerstown) 2020;21(9):648–653; doi: 10.2459/JCM.0000000000001011 32628426

[24] Braunschweig F, Anker SD, Proff J, et al. Remote monitoring of implantable cardioverter-defibrillators and resynchronization devices to improve patient outcomes: Dead end or way ahead? Europace 2019;21(6):846–855; doi: 10.1093/europace/euz011 30903152 PMC6545502

[25] Morgan JM, Kitt S, Gill J, et al. Remote management of heart failure using implantable electronic devices. Eur Heart J 2017;38(30):2352–2360; doi: 10.1093/eurheartj/ehx227 28575235 PMC5837548

[26] Husser D, Christoph Geller J, Taborsky M, et al. Remote monitoring and clinical outcomes: Details on information flow and workflow in the IN-TIME study. Eur Heart J Qual Care Clin Outcomes 2019;5(2):136–144; doi: 10.1093/ehjqcco/qcy031 30016396 PMC6440440

[27] Zanotto G, Melissano D, Baccillieri S, et al. Intrahospital organizational model of remote monitoring data sharing, for a global management of patients with cardiac implantable electronic devices: A document of the Italian Association of Arrhythmology and Cardiac Pacing. J Cardiovasc Med (Hagerstown) 2020;21(3):171–181; doi: 10.2459/JCM.0000000000000912 32004241

[28] Odendaal WA, Anstey Watkins J, Leon N, et al. Health workers' perceptions and experiences of using mHealth technologies to deliver primary healthcare services: A qualitative evidence synthesis. Cochrane Database Syst Rev 2020;3(3):CD011942; doi: 10.1002/14651858.CD011942.pub2 32216074 PMC7098082

